# Insights into the clinical and immunological significance of anti-α-fodrin antibodies in systemic lupus erythematosus

**DOI:** 10.1515/rir-2025-0007

**Published:** 2025-04-02

**Authors:** Fernanda Espinosa-Bautista, Varna Ramos-Rosillo, Yadira Vazquez-Panchos, Fernanda Bocanegra-Zamora, Valentin Jimenez-Rojas, Ricardo Márquez-Velasco, Luis M. Amezcua-Guerra

**Affiliations:** Immunology Department, Instituto Nacional de Cardiología Ignacio Chávez, Mexico City, Mexico; Health Care Department, Universidad Autónoma Metropolitana Xochimilco, Mexico City, Mexico

Dear Editor,

Alpha (α)-fodrin, a non-erythroid homolog of spectrin, plays a role in maintaining the structural stability of cell membranes. During apoptosis, α-fodrin undergoes enzymatic cleavage by caspase-3, generating smaller fragments that act as neoantigens.^[1]^ Anti-α-fodrin antibodies are found in autoimmune disorders, including primary Sjögren’s syndrome (70%) and systemic lupus erythematosus (SLE; 10%–30%), as well as in a minority of healthy individuals (~2%).^[2,3]^ Experimental studies in animal models have demonstrated that immunization with α-fodrin induces lympho-cytic infiltration in salivary glands, while immunization with human Ro antigen results in the production of anti-α-fodrin antibodies, suggesting a potential shared intermolecular epitope between Ro and α-fodrin.^[4,5]^ However, the association between anti-α-fodrin antibodies and SLE-related clinical or immunological manifestations remains insufficiently investigated and is often overlooked in studies where SLE patients serve only as controls.

Our study aimed to elucidate the association between anti-α-fodrin antibodies and clinical as well as immunological perturbations in SLE, particularly their relationship with anti-Ro/anti-Ro52 antibodies and chemokine levels.

The study was approved by the local ethics committee (protocol number 16–960) and participants provided written informed consent. Thirty adult SLE patients (86% female; median age: 41 [19–63] years) without sicca symptoms, xero-stomia, xerophthalmia, or Sjögren’s syndrome participated in the study. Disease activity was assessed using the systemic lupus erythematosus disease activity index-2000 (SLEDAI-2K) score, with organ involvement categorized based on predefined criteria detailed elsewhere.^[6]^ Anti-α-fodrin IgA and IgG antibodies (cut-off ≥10 U/mL), as well as anti-Ro and an-ti-Ro52 antibodies (≥25 U/mL) (Orgentec; Mainz, Germany), were measured by Enzyme linked immunosorbent assay (ELISA). Positivity for anti-α-fodrin antibodies was defined by the detection of either IgA or IgG antibodies. Serum chemo-kines were measured using multiplexed bead-based assays (ThermoFisher Scientific; Minneapolis, United States).

Six patients tested positive for anti-α-fodrin IgA antibodies, while two tested positive for anti-α-fodrin IgG antibodies. Table 1 summarizes the main clinical and laboratory findings. No significant differences in demographics, comorbidities, disease severity, organ involvement, or medication usage were observed between antibody-positive and antibody-negative patients. Similarly, there were no differences in serum positivity for other SLE-associated antibodies. Median levels of anti-Ro (0, 3–35 U/mL *vs*. 0, 0–108 U/mL; *P* = 0.47) and anti-Ro52 (0, 0–21 U/mL *vs*. 0, 0–97 U/mL; *P* = 0.86) antibodies were similar between the groups.

**Table 1:** Clinical and laboratory data according to anti-α-fodrin antibody status

		Anti-α-fodrin (+) (*n* = 8)	Anti-α-fodrin (-) (*n* = 22)	*P*
Female, *n*(%)		7 (87)	19 (86)	>0.99
Age, years		39 (25-62)	41 (19-63)	0.95
Disease duration, years		6 (2-25)	9 (1-20)	0.63
Hypertension, *n*(%)		2 (25)	8 (36)	0.68
Diabetes, *n*(%)		1 (12)	2 (9)	>0.99
Antiphospholipid syndrome-	*n*(%)	1 (12)	7 (31)	0.39
SLEDAI-2K score		4 (2-9)	5 (2-13)	0.29
Organ involvement, *n*(%)				
Musculoskeletal		2 (25)	2 (9)	0.28
Renal		1 (12)	9 (40)	0.21
Hematological		1 (12)	2 (9)	>0.99
Neuropsychiatric		0	0	>0.99
Vasculitis		0	1 (4)	>0.99
Serositis		1 (12)	2 (9)	>0.99
Drug therapy, *n*(%)				
Prednisone		3 (37)	12 (54)	0.68
Azathioprine		3 (37)	5 (22)	0.64
Cyclophosphamide		3 (37)	3 (13)	0.30
Mofetil mycophenolate		2 (25)	5 (22)	>0.99
Antimalarials		7 (87)	17 (77)	>0.99
Calcineurin inhibitors		0	2 (9)	>0.99
Laboratory studies				
Leukocytes, ×10^3^ /mm^3^3		5.7 (2.9-6.9)	5.1 (2.7-12.1)	0.54
Lymphocytes, ×10^3^ /mm^3^		1.3 (0.7-2.4)	1.6 (0.3-6.4)	0.95
Platelets, ×10^3^ /mm^3^		234 (141-389)	216 (32-400)	0.41
Hemoglobin, g/dL		14.0 (9.7-16.2)	14.0 (11.2-16.4)	0.88
Serum creatinine, mg/dL		0.6 (0.6-0.9)	0.8 (0.5-1.4)	0.10
C3 complement, mg/L		100 (41-129)	89 (23-136)	0.61
C4 complement, mg/L		16 (3-28)	15 (1-40)	0.68
C-reactive protein, mg/L		1.2 (0.4-11.5)	2.4 (0.5-11.9)	0.56
Anti-Ro (+), *n*(%)		1 (12)	7 (31)	0.39
Anti-Ro52 (+), *n*(%)		0	4 (18)	0.55
Anti-La (+), *n*(%)		1 (12)	2 (9)	>0.99
Anti-dsDNA (+), *n*(%)		5 (62)	18 (81)	0.34
Anti-Sm (+), *n*(%)		4 (50)	5 (22)	0.19
Serum chemokines				
CCL11/eotaxin		54 (30-132)	91 (23-339)	0.02
CCL2/MCP-1		136 (36-394)	379 (107-2211)	< 0.01
CCL5/RANTES		118 (88-2500)	165 (56-8870)	0.60
CCL3/MIP-1α		63 (11-288)	87 (21-340)	0.51
CCL4/MIP-1β		275 (148-364)	334 (170-716)	0.23
CXCL10/IP-10		54 (39-151)	93 (23-741)	0.39
CXCL12/SDF-1		687 (437-1213)	723 (297-2375)	0.47
CXCL8/interleukin 8		15 (5-37)	15 (4-109)	0.57

Categorical variables are presented in percentages, and differences were assessed using Fisher’s exact test. Numerical variables are represented by medians (minimum-maximum range), and differences were analyzed using the Mann-Whitney *U* test. Significant *P* values are highlighted in bold. SLEDAI-2K, Systemic Lupus Erythematosus Disease Activity Index 2000; CCL11, C-C motif ligand 11; CCL2, C-C motif ligand 2; MCP-1, monocyte chemoattractant protein 1; CCL-5, C-C motif ligand 5; RANTES, regulated on activation, normal T-cell expressed and secreted; CCL-3, C-C motif ligand 3; MIP-1α, macrophage inflammatory protein 1-alpha; CCL-4, C-C motif ligand 4; MIP-1β, macrophage inflammatory protein 1-beta; CXCL10, C-X-C motif ligand 10; IP-10, interferon gamma-induced protein 10; CXCL12, C-X-C motif ligand 12; SDF-1, stromal cell-derived factor 1; CXCL8, C-X-C motif ligand 8.

**Figure 1:**
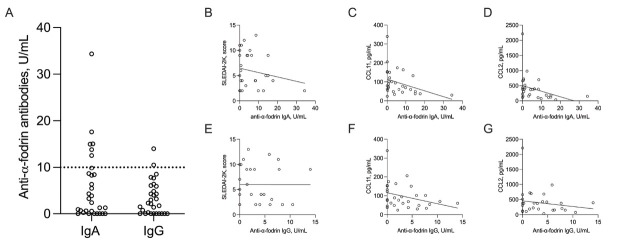
Anti-α-fodrin antibodies in systemic lupus erythematosus (SLE). Panel A presents the levels of anti-α-fodrin IgA and IgG antibodies in SLE patients, with a dotted line marking the positivity threshold. Panel B shows the absence of a significant correlation between anti-α-fodrin IgA levels and disease activity. Panel C illustrates a significant inverse correlation between anti-α-fodrin IgA levels and serum CCL11/eotaxin levels, while panel D depicts a similar correlation with CCL2/MCP-1 levels. In contrast, panels E–G demonstrate no significant associations between anti-α-fodrin IgG levels and disease activity, CCL11/eotaxin, or CCL2/MCP-1 levels, respectively.

The median levels of anti-α-fodrin IgA antibodies were 2.8 (0–34.3) U/mL, and for IgG antibodies, the median was 2.2 (0–14.0) U/mL (Figure 1–A). Disease activity analysis indicated that 26% of patients had quiescent disease, 66% had mild to moderate activity, and 6% had high activity. Nonetheless, no correlation was observed between SLEDAI-2K scores and anti-α-fodrin IgA (Spearman’s rho-0.02 [-0.39 to 0.34]; *P* = 0.89. Figure 1–B) or IgG (rho-0.04 [-0.40 to 0.33]; *P* = 0.81. Figure 1–E) antibody titers.

Chemokine analysis revealed that anti-α-fodrin-positive patients exhibited lower levels of C-C motif ligand 11 (CCL11)/eotaxin (54, 30–132 ng/L *vs*. 91, 23–339 ng/L; *P* = 0.02) and C-C motif ligand 2 (CCL2)/monocyte chemoattractant protein 1 (MCP-1) (136, 36–394 *vs*. 379, 107–2211 ng/L; *P* < 0.01) compared to antibody-negative patients. Other chemokine levels were similar between the groups (Table 1). Furthermore, levels of anti-α-fodrin immunoglobulin (IgA) antibodies were inversely correlated with CCL11/eotaxin (rho-0.38 [-0.66 to-0.02]; *P* = 0.03. Figure 1–C) and CCL2/MCP-1 (rho-0.39 [-0.66 to-0.03]; *P* = 0.02. Figure 1–D). Conversely, anti-α-fodrin IgG antibodies showed no significant correlation with CCL11/eotaxin (rho-0.32 [-0.62 to 0.04]; *P* = 0.07. Figure 1–F) or CCL2/MCP-1 (rho-0.03 [-0.39 to 0.34]; *P* = 0.86. Figure 1–G).

This study provides novel insights into the clinical and immunological roles of anti-α-fodrin antibodies in SLE. Contrary to initial expectations, no significant associations were observed between anti-α-fodrin antibodies and disease severity, organ involvement, or anti-Ro/anti-Ro52 antibodies in SLE patients. These findings contrast with animal models of Sjögren’s syndrome, where a connection between anti-α-fodrin and anti-Ro antibodies has been suggested.^[4,5,7]^ Interestingly, our findings reveal a potential link between anti-α-fodrin antibodies and reduced levels of certain chemokines, suggesting a possible protective role in modulating the inflammatory response triggered by α-fodrin-induced apoptosis. This mechanism aligns with other cases of antibody-mediated regulation of harmful proteins in SLE, such as anti-cytokine autoantibodies that neutralize excessive cytokine activity and mitigate tissue damage.^[8]^ A plausible mechanism is that anti-α-fodrin antibodies may sequester free α-fodrin, preventing its activation of caspase-3 and subsequent apoptosis. This could reduce the production of chemokines like CCL2/MCP-1 and CCL11/eotaxin, which are crucial for recruiting immune cells such as monocytes and granulocytes.^[9,10]^

Although the small sample size and cross-sectional design limit the ability to draw definitive conclusions, our study failed to establish a clear association between anti-α-fodrin antibodies and anti-Ro/anti-Ro52 antibodies, disease severity, or organ-specific involvement in SLE. Nevertheless, our findings suggest a potential mechanistic role for these antibodies in regulating chemokine-driven inflammation.
